# Strain-dependent variations in replication of European clade 2.3.4.4b influenza A(H5N1) viruses in bovine cells and thermal inactivation in semi-skimmed or whole milk

**DOI:** 10.2807/1560-7917.ES.2024.29.30.2400436

**Published:** 2024-07-25

**Authors:** Diana I Palme, Juliane Lang, Dajana Helke, Maryna Kuryshko, Elsayed M Abdelwhab

**Affiliations:** 1 Institute of Molecular Virology and Cell Biology, Friedrich-Loeffler-Institut, Federal Research Institute for Animal Health, Greifswald-Insel Riems, Germany

**Keywords:** influenza A virus, H5N1 clade 2.3.4.4b, cow, milk, thermostability, bovine cells

## Abstract

We investigated the thermostability of four European avian influenza A(H5N1) viruses in whole and semi-skimmed milk and their replication in bovine kidney and lung cells amid the current influenza A(H5N1) dairy cattle outbreak in the United States. Results showed strain-dependent differences in thermal inactivation, particularly in whole milk, and variable replication efficacy in lung cells. These findings support assessing the inactivation of European H5N1 viruses in milk and their replication in bovine cells, aiding biosafety protocols and public health measures.

Cattle were once thought to be resilient to avian influenza virus (AIV) infections [1], until the recent widespread influenza A(H5N1) virus infections in dairy cattle in several states in the Unites Stares (US) since March 2024 [2]. Surprisingly, high viral loads were found in the milk of infected cows, but not in the respiratory tract [3]. Consumption of unpasteurised influenza A(H5N1)-contaminated milk represents a new niche of possible public health concern for an avian virus. Cats on the dairy farms [3] and laboratory mice got infected and died after drinking influenza A(H5N1)-contaminated unpasteurised colostrum and milk [4]. To date, four dairy farm workers have been reported with influenza A(H5N1)-associated conjunctivitis and upper respiratory influenza-like illness [5]. 

Little is known about the efficiency of H5N1 clade 2.3.4.4b, particularly non-US isolates, to replicate in bovine cells and remain infectious in milk with different fat contents. Here we investigated the effect of different fat contents on the thermostability and duration of heat inactivation of H5N1 influenza viruses and assessed the replication of recent German H5N1 viruses of clade 2.3.4.4b in bovine kidney and lung cells.

## Thermal inactivation of influenza A(H5N1) viruses in semi-skimmed and whole milk 

Four influenza A(H5N1) viruses isolated in Germany were used in this study. These included three recent clade 2.3.4.4b viruses A/chicken/Germany/AI04286/2022 (designated H5N1-chicken), A/wood pigeon/Germany-NW/AI00951/2022 (H5N1-Pigeon; accession number EPI_ISL_10261376) and A/red knot/AI000616/2022 (H5N1-Knot; accession number EPI_ISL_18006920), as well as a clade 2.2.2 A/swan/Germany/R65/2006 (designated H5N1-Swan; accession number EPI_ISL_10142). In addition, the historic A/turkey/England/384/79 (H10N4) virus was used as a control. 

**Figure 1:**
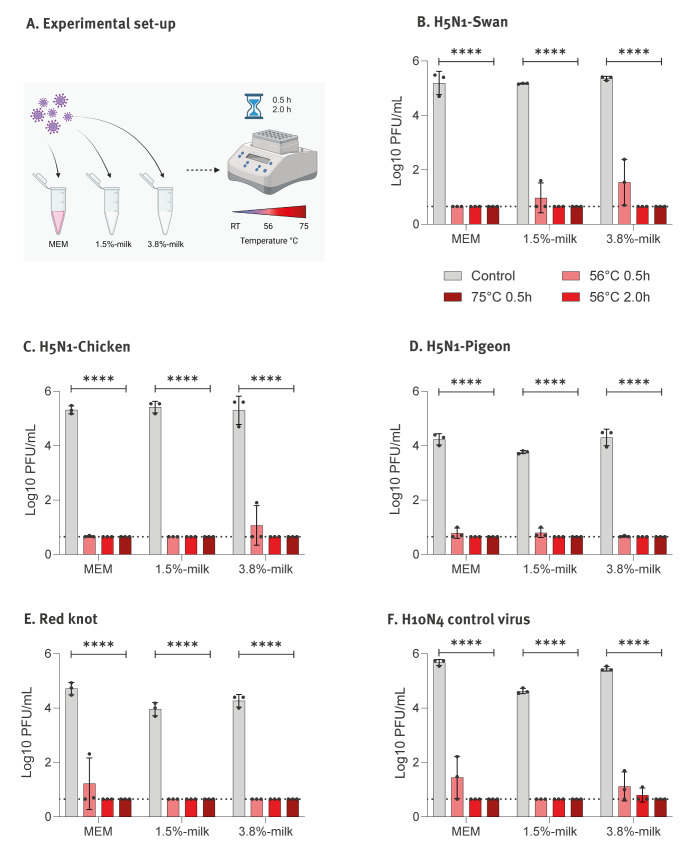
Thermal inactivation of influenza A(H5N1) viruses in semi-skimmed and whole milk

MEM: minimal essential medium; PFU: plaque-forming units; RT: room temperature.

We assessed the thermostability of the viruses in commercial semi-skimmed milk (1.5% fat), and whole milk (3.8% fat) compared with cell culture minimal essential medium (MEM). The milk and MEM were spiked with ca 100,000 plaque-forming units (PFU) of virus and incubated at room temperature (20–22 °C) for 60 min. Subsequently, samples were heat-inactivated at 56 °C or 75 °C using a heat block (Figure 1A). These temperatures are commonly used for inactivating biological fluids such as serum, blood or allantoic fluid in biosafety level 3 (BSL3) facilities. The heat inactivation duration was set to 0.5 h for both temperatures and 2 h specifically at 56 °C, reflecting our BSL3 laboratory protocol. We included control samples of virus-containing milk and medium without heat treatment.

Thermostability of each virus was evaluated by heat inactivation at different conditions: 30 min at 56 °C, 120 min at 56 °C or 30 min at 75 °C. Control samples were left untreated and titrated 1 h after spiking with viruses. Experiments were conducted in triplicate and viral titres determined promptly. Dotted lines indicate the detection limit of the plaque assays. Statistical significance was assessed using two-way ANOVA for each treatment compared with the control. Asterisks denote significant differences (**** ≤ 0.0001). Statistical analysis was conducted using GraphPad Prism, version 9.2.0 (www.graphpad.com). Figures were created using BioRender.

Heat inactivation at 75 °C for 30 min or 56 °C for 120 min effectively reduced the infectivity of all viruses below the detection limit of the plaque assays (Figure 1B–F) [6]. However, heat inactivation for 30 min at 56 °C did not completely eliminate the infectivity of the tested AIV: Despite reduced titres, H10N4, H5N1-Swan and H5N1-Chicken retained infectivity in whole milk, and H5N1-Swan and H5N1-Pigeon were detectable in semi-skimmed milk (Figure 1). In addition, infectious H10N4 and, to a lesser extent, H5N1-Knot and H5N1-Pigeon viruses were detected in virus-spiked MEM (Figure 1). Overall, thermostability varied by virus strain and fat content of the milk. Complete virus inactivation required heat treatment at 56°C for 120 minutes.

## Replication of influenza A(H5N1) viruses in bovine cell culture

**Figure 2:**
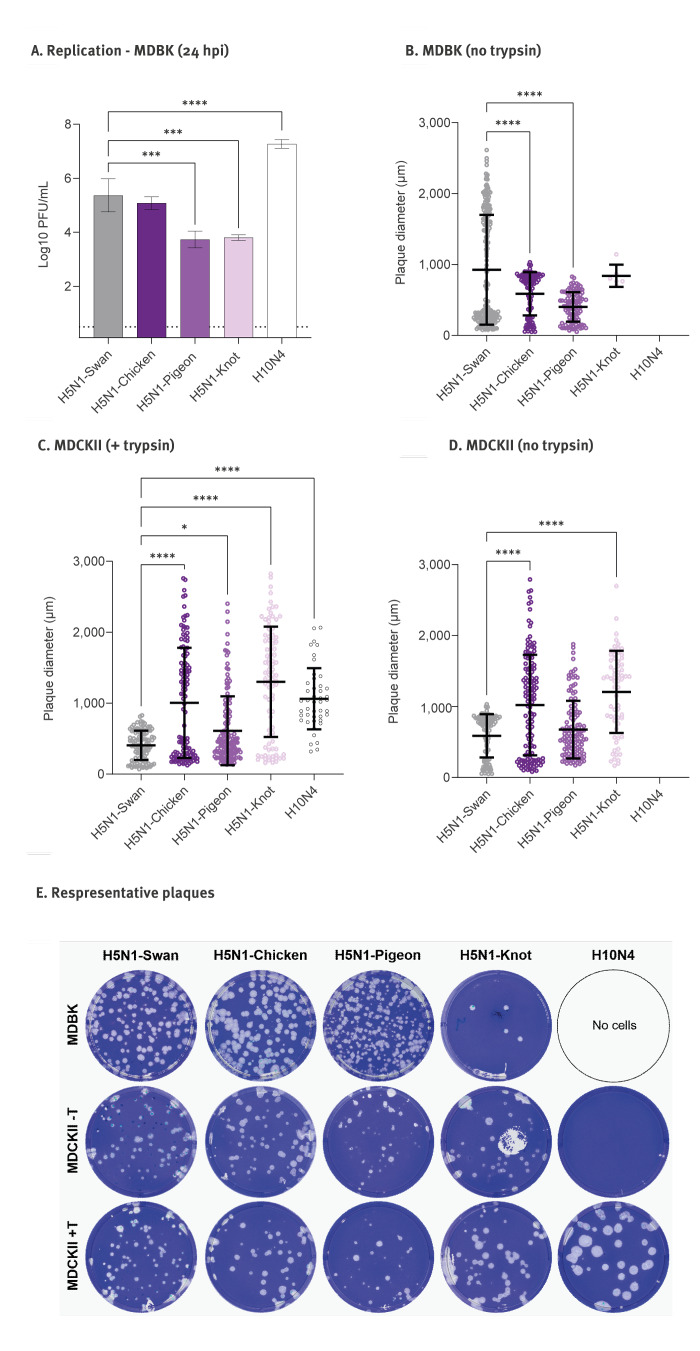
Replication and cell-to-cell spread of four influenza A(H5N1) virus strains in bovine kidney cells

MDCKII: Madin–Darby canine kidney type II; MDBK: Madin­–Darby bovine kidney; hpi: hours post infection; PFU: plaque-forming units; +T: with trypsin; −T: without trypsin.

Viral replication was assessed in Madin–Darby bovine kidney (MDBK) cells infected with a multiplicity of infection (MOI) of 0.001 for 24 h. All H5N1 viruses replicated in MDBK cells (Figure 2A), while H10N4 did not induce detectable titres without trypsin (data not shown). The levels of H5N1-Knot and H5N1-Pigeon were ca 10 times lower than those of H5N1-Swan and H5N1-Chicken (Figure 2A). We further assessed the cell-to-cell spread in MDBK cells relative to Madin–Darby canine kidney type II (MDCKII) cells by measuring plaque diameters. The intercellular spread of H5N1 clade 2.3.4.4b viruses in MDBK cells was notably more homogeneous compared with clade 2.2.2 H5N1-Swan (Figure 2B). In contrast, MDCKII cells showed a mixed population of plaques, indicating a more heterogeneous spread (Figure 2C–E).

Panel A: An MDBK cell line was infected with indicated viruses at a multiplicity of infection of 0.001 for 1 h. Data are presented as means ± standard deviations of two independent experiments of triplicates. TPCK-treated trypsin was added to H10N4 virus. Viral titres were determined using a standard plaque assay in MDCKII cells. The dotted line indicates the detection limit of the plaque assay.

Panels B–D: Intercellular spread of indicated viruses, assessed by plaque assay in MDBK cells compared with standard MDCKII cells in the presence or absence of trypsin. Cells were stained with crystal violet containing formalin at 72 h after infection, and plaque diameters were measured using NIKON software under a microscope. Data are presented as means ± standard deviation.

Panel E: Representative plaques visualised with crystal violet staining. The MDBK cells showed high sensitivity to trypsin, leading to detachment of all cells after infection with H10N4 (upper right well).

**Figure 3:**
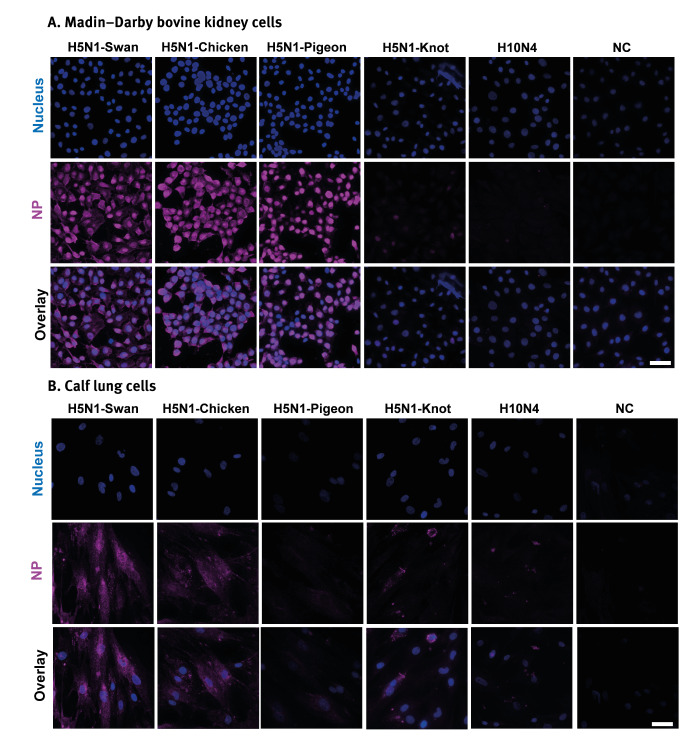
Detection of influenza A(H5N1) nucleoprotein of four different virus strains in bovine cells

NC: negative control without infection; NP: nucleoprotein.

To visualise virus infection, MDBK and calf lung (KLU) cells were infected at an MOI of 0.1 and subjected to standard immunofluorescence procedures (Figure 3). The H5N1-Swan and H5N1-Chicken viruses demonstrated higher efficacy in infecting both MDBK (Figure 3A) and KLU cells (Figure 3B) compared with H5N1-Knot and H10N4. The detection of influenza A(H5N1) nucleoprotein in KLU cells was notably lower than in MDBK cells, indicating that these viruses are less effective at infecting lung cells compared to kidney cells. Interestingly, H5N1-Pigeon was detected at higher levels in MDBK cells than H5N1-Knot, which was barely detected. In lung cells, H5N1-Pigeon and H5N1-Knot were scarcely detected (Figure 3B).

Representative immunostaining after infection at a multiplicity of infection of 0.1. At 8 h post-infection. Cells were subjected to indirect immunofluorescence assay with rabbit anti-nucleoprotein (magenta) polyclonal antibody. Nuclei were stained with Hoechst (blue). Scale bars: 15 µm. We added 1% allantoic fluid instead of trypsin to H10N4 virus to avoid detachment of the cells.

**Figure 4:**
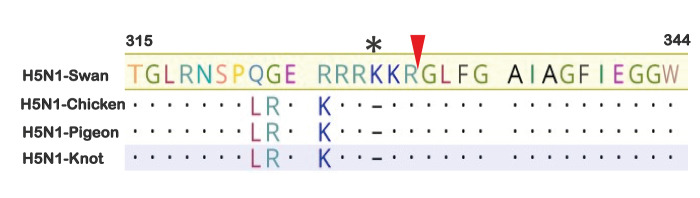
Deduced amino-acid sequence of the haemagglutinin cleavage site of four influenza A(H5N1) viruses used in this study

We compared the sequence of the HA cleavage site as a main determinant for virus replication. All three 2.3.4.4b viruses in this study exhibited a deletion of a basic amino acid in the HA cleavage site, in contrast to the clade 2.2.2 H5N1-Swan virus (Figure 4). 

Alignment of the peptide spanning the haemagglutinin cleavage site (indicated by a red triangle), with the characteristic deletion (*) of lysine (K) specific to clade 2.3.4.4b viruses.

## Discussion

During the current H5N1 outbreak in the US, raw cow milk has been identified as a source of influenza virus infection among mammals near infected dairy cows. In addition, bovine A(H5N1) and human A(H1N1)pdm09 influenza viruses retained infectivity on milking machines for up to 3 h [7]. It is known that pasteurisation of milk at 63 °C for 30 min or, alternatively, heating to 72–154 °C for a few seconds, inactivates a wide range of pathogens and ensures safety for human consumption [8-10]. Studies have shown that American and Asian influenza viruses are effectively inactivated at pasteurisation temperatures. It remains unclear if milk with varying fat contents presents a unique medium for influenza virus infectivity compared with allantoic fluid or serum samples. Our study demonstrated that all viruses were undetectable after treatment at 75 °C for 30 min, consistent with recent findings on American and Chinese H5N1 influenza viruses [4,11,12]. Interestingly, while viral titres significantly decreased at 56 °C, residual infectious virus was detected after 30 min in whole milk (three strains), semi-skimmed milk (one strain) and MEM (two strains). These findings suggest thermostability may vary by virus strain, and whole milk could impact the stability of certain influenza viruses. Similar strain-dependent variations were seen in Chinese virus-spiked milk [12]. To ensure complete inactivation of A(H5N1) viruses and thus biosafety under laboratory conditions, higher temperatures should be considered.

Furthermore, all H5N1 viruses and H10N4 (in the presence of trypsin), replicated efficiently in bovine cells, whereas pigeon and red knot viruses exhibited lower titres. This indicates that certain H5N1 viruses can replicate effectively in bovine lung and kidney cells without prior adaptation. The reasons for the cell and virus dependency to infect bovine cells are not clear, and additional studies are needed to understand the distribution of sialic acid receptors on both cell types and receptor binding specificity/affinity of these viruses.

Our study has several limitations. Firstly, AIV-contaminated milk used in laboratory settings may not fully replicate natural milk conditions. Understanding how fat influences virus stability can help improve safety measures particularly in rural areas where standard pasteurisation conditions may not be met. Secondly, the heating times used in this study, though relevant for laboratory biosafety and potentially for sterilising contaminated farm equipment, exceed the standard pasteurisation procedures employed in the dairy industry. This difference in heating protocols may impact the generalisability of our findings to practical dairy processing scenarios. It is worth mentioning that in Europe, ultra-high-temperature (UHT) treatment is used instead of pasteurisation; it heats milk to 138–150 °C for 1–2 sec [13]. Our findings provide valuable insights into AIV replication in cell culture; however, they may not directly correlate with the infection dynamics in live cows, underscoring the need for in vivo studies to validate our results. Despite these limitations, the strain-dependent and cell-dependent variations observed in our study highlight the potential complexity of the current dairy cattle outbreak and emphasise the necessity for further research to fully understand these dynamics and their broader implications.

## Conclusion

This study demonstrates that non-US influenza A(H5N1) viruses can also infect and replicate in bovine kidney and lung cells, albeit with varying titres, spread and infectivity. Some viruses remained infectious after exposure to 56 °C for 30 min, particularly in whole milk. Therefore, considering strain dependency is crucial when determining virus inactivation protocols at this temperature for biosafety reasons.
